# Analysis of 27 β-Blockers and Metabolites in Milk Powder by High Performance Liquid Chromatography Coupled to Quadrupole Orbitrap High-Resolution Mass Spectrometry

**DOI:** 10.3390/molecules24040820

**Published:** 2019-02-25

**Authors:** Jian-Qiao Cheng, Tong Liu, Xue-Mei Nie, Feng-Ming Chen, Chuan-Sheng Wang, Feng Zhang

**Affiliations:** 1 Institute of Food Safety, Chinese Academy of Inspection & Quarantine, Beijing 100176, China; 13352479167@163.com (J.-Q.C.); liutongyes@163.com (T.L.); niexuemei_00@163.com (X.-M.N.); chenfengmingok@163.com (F.-M.C.); 2 College of Applied Chemistry, Shenyang University of Chemical Technology, Shenyang 110142, China; wchsh18@163.com

**Keywords:** β-blockers, metabolites, milk powder, Q-Orbitrap

## Abstract

This paper presents an application of high performance liquid chromatography coupled with quadrupole orbitrap high-resolution mass spectrometry (HPLC-Q-Orbitrap HRMS) for the analysis of 27 β-blockers and metabolites in milk powder. Homogenized milk power samples were extracted by acetonitrile and purified by using Oasis PRiME HLB solid-phase extraction cartridges. The Ascentis^®^ C8 chromatographic column was used to separate the analytes. The quantification was achieved by using matrix-matched standard calibration curves with carazolol-d_7_ and propranolol-d_7_ as the internal standards. The results show an exceptional linear relationship with the concentrations of analytes over wide concentration ranges (0.5–500 μg kg^−1^) as all the fitting coefficients of determination r^2^ are > 0.995. All the limits of detection (LODs) and quantitation (LOQs) values were within the respective range of 0.2–1.5 μg kg^−1^ and 0.5–5.0 μg kg^−1^. Overall average recoveries were able to reach 66.1–100.4% with the intra- and inter-day variability under 10%. This method has been successfully applied to the screening of β-blockers and metabolites in commercial milk powders. At the same time, the corresponding characteristic fragmentation behavior of the 27 compounds was explored. The characteristic product ions were determined and applied to the actual samples screening.

## 1. Introduction

β-blockers (BBS) are structurally analogous to the catecholamines, which can act as non-specific β-adrenergic receptor blocking agents. They play an extremely important role in the treatment of cardiovascular diseases such as coronary heart disease, hypertension, arrhythmia and cardiac insufficiency. However, improper use of β-blockers can cause an increase of myocardial oxygen consumption, vascular resistance, oxygen free radicals and myocardial cell apoptosis, etc. [1]. β-blockers are usually used in animals to reduce morbidity and mortality during transportation (to the slaughterhouse or livestock farm), mating, childbirth and in other stressful situations. Such stress usually results in a poor quality of meat, or even in the premature death of the animal [2,3,4]. The illegal use of β-blockers gives rise to drug residues in edible animal tissue, which can be metabolized in the body. Several metabolites of β-blockers are pharmacologically active and also harmful to the body. For example, 4-hydroxyphenyl carvedilol (the metabolite of carvedilol) exhibits an approximately thirteen-fold higher-adrenoreceptor blocking potency compared to carvedilol itself [5]. In order to protect public health, many countries and organizations began to establish regulations. For example, carazolol has maximum residue limits (MRLs) in animal-based foods. The European Union and the International Codex Alimentarius Commission have asked for MRLs of carazolol in edible animal tissues, with MRLs of 25 μg kg^−1^ for porcine kidney and 15 μg kg^−1^ for bovine kidney. The European Union has also asked for a maximum residue limit of 1.0 μg kg^−1^ of carazolol in the milk powder [1,6]. However, no restrictions have been placed upon their metabolites (4-hydroxyphenyl carvedilol, etc.). Due to the fact that eating food containing high levels of carazolol and other β-blockers can be harmful to consumer health (especially to infants and children), control of β-blockers is required [3]. Therefore, it is necessary to establish a high-throughput analytical method for β-blockers and their metabolites.

Many approaches for the detection of β-blockers have been reported, such as enzyme-linked immunosorbent assay (ELISA) [7], gas chromatography coupled with mass spectrometry (GC-MS) [8,9,10,11], liquid chromatography with fluorescence detection (LC-UV) [12], and liquid chromatography mass spectrometry (LC-MS) [13,14,15,16,17]. Although these methods play important roles in the detection of β-blockers, they also have some drawbacks. For example, the quantification of the ELISA method is not accurate and the operation is troublesome, and GC-MS involves derivative steps before chromatographic separation, which are time-consuming and which increase the possibility of contamination [18]. And the LC-UV method is limited by poor sensitivity, so it cannot meet the requirements for ultra-trace analyses. LC-MS was the most widely used method for the qualitative and quantitative detection of β-blockers multiple residues. Liquid chromatography is generally used, coupled with a low resolution mass spectrometry (LRMS) analyzer such as triple-quadrupole (QqQ). Orbitrap is the newest HRMS analyzer. Most identification and determination studies of β-blockers were undertaken using the LTQ Orbitrap (linear ion trap quadrupole Orbitrap high resolution mass spectrometry), achieving LODs below 2 μg kg^−1^ and 5 ng mL^−1^ [19,20]. The Q-Orbitrap (Q-Exactive^TM^, hybrid quadrupole-orbitrap mass spectrometer, Thermo Fisher Scientific, Bremen, Germany) combines high-performance quadrupole precursor selection with high resolution and accurate mass (HR/AM) Orbitrap detection, which has great potential to avoid both false positive and negative results in residue analyses. Compared with the LTQ Orbitrap, the Q-Orbitrap has higher sensitivity, and its use has become widespread in the confirmation and quantification of drugs residues in food [21,22]. In addition, it can realize real-time positive and negative switches; therefore, the time spent on the preparation process and method optimization is significantly reduced. However, analysis of β-blockers and their metabolites using the Q-Orbitrap has not been reported.

In this study, a high-throughput, high performance liquid chromatography coupled to quadrupole Orbitrap high-resolution mass spectrometry (HPLC-Q-Orbitrap HRMS) has been developed for the screening of 27 analytes, including 21 β-blockers and 6 metabolites in milk powder samples. In addition to this, the corresponding characteristic fragmentation behavior and the product ions of the 27 compounds are described in detail. They will provide a basis for the target-free screening of these drugs and the identification markers of the newly-emerging β-blockers residues.

## 2. Results and Discussion

### 2.1. LC Parameters Optimization

**Figure 1:**
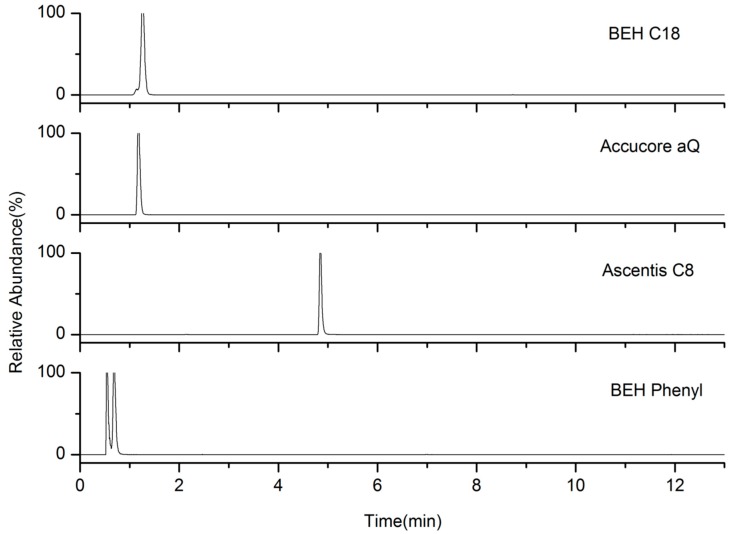
The effect of the columns on the chromatographic separation of hydroxyatenolol.

Chromatographic conditions were studied in order to obtain the best separation and retention for the compounds. Four HPLC columns (Waters ACQUITY UPLC^®^ BEH C18 (1.7 μm, 50 × 2.1 mm, Milford, MA, USA), Thermo Accucore aQ (2.6 μm, 150 × 2.1 mm, Bellefonte, PA, USA), ALDRICH Ascentis^®^ C8 (3 μm, 10 cm × 4.6 mm, Bellefonte, PA, USA), Waters ACQUITY UPLC^TM^ BEH Phenyl (1.7 μm, 50 × 2.1 mm, Milford, MA, USA) were evaluated in (0.1% FA) H_2_O-MeCN in their appropriate gradient elution at 0.5 mL min^−1^. Similar separation performances for total analytes were observed by the first three columns; however, the Ascentis^®^ C8 column provided better shape and retention for hydroxyatenolol, as shown in Figure 1. Therefore, the Ascentis^®^ C8 column was chosen.

**Figure 2:**
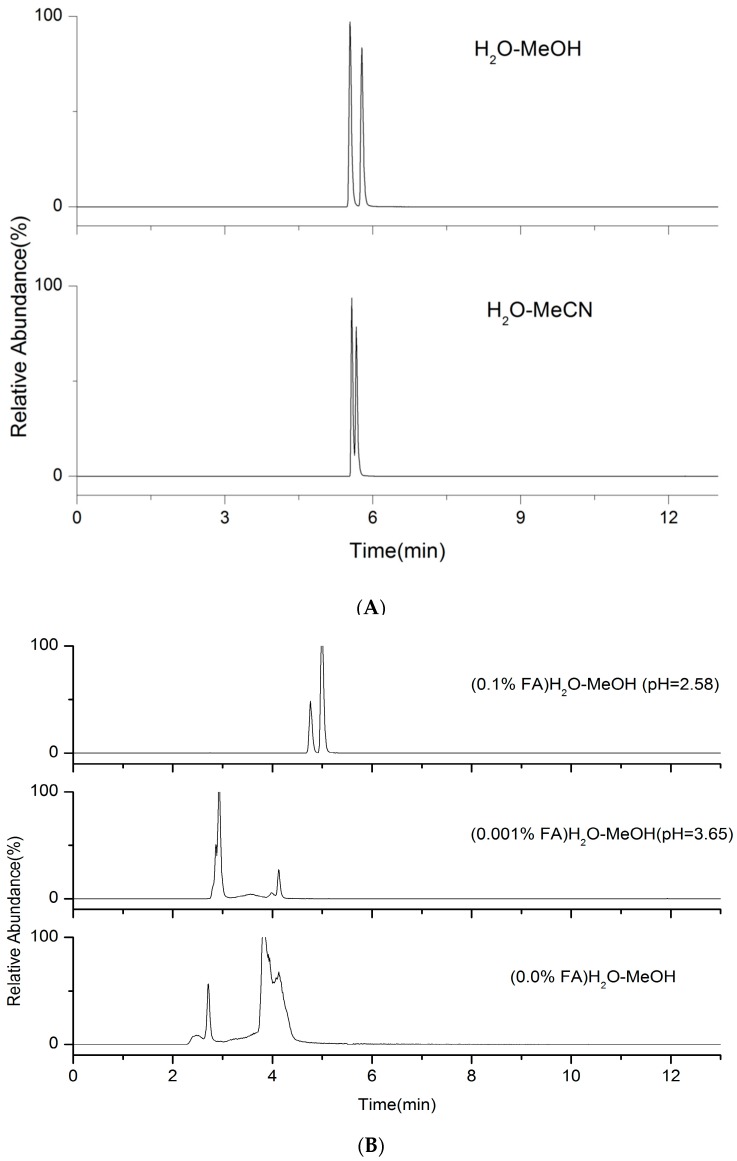
The effect of different mobile phases on the chromatographic separation of the isomers of atenolol and practolol. (**A**) Different organic solvents (atenolol:practolol = 2:1); (**B**) Different concentrations of FA in water phase (atenolol:practolol = 1:1).

Several mobile phases were tested using MeCN or MeOH as an organic solvent and water as a polar solvent with FA addition (from 0% to 0.5%). As shown in Figure 2A, the mixture of MeOH and water showed higher responses and better separation for isomers of practolol and atenolol. The proton donor tendency of MeOH contributed to the formation of positive adducts. The better volatility and lower surface tension of MeOH can also improve desolvation of the droplets. Therefore, MeOH was chosen as the organic solvent. On the other hand, the addition of FA could improve the phenomenon of peak tailing and the response, which is probably because the excess silanols in the stationary phase combine with the acid rather than with their targets. The retention time of isomers of atenolol and practolol were greatly affected by pH, so a good chromatographic separation could not obtained until 0.1% FA was added (mobile phase pH = 2.58) (Figure 2B). Therefore, 0.1% FA-H_2_O was chosen as the water phase.

In addition, injection volumes of 2 μL to 5 μL were evaluated using the aforementioned conditions. Taking the isomers of atenolol and practolol as examples, the experimental results showed that the separation factor decreased from 1.41 to 0.77 with increasing injection volume. So, 2 μL was chosen as the experimental injection volume. 

Furthermore, several elution gradient profiles were also optimized to obtain better chromatographic separation and less analysis time (within 15 min). Other parameters (flow rate, column temperature) were also characterized to achieve better target separation and peak shapes. Under these conditions (see Section 3.2), the retention times (RT) of these 27 analytes were constant, and ranged ranging from 3.85 min to 9.01 min.

### 2.2. Optimization of the Mass Spectrometric Parameters

The optimization of MS parameters was performed by infusing a standard solution of 100 μg L^−1^ of each β-blockers in methanol-water (50:50, *v*/*v*) as the mobile phase under Full scan mode (Full MS). The precursor ions were selected in both positive and negative modes. Consistent with previous report [2], β-blockers and metabolites tend to form [M + H]^+^ adduct ions in positive mode. The Full MS/ddMS^2^ scan mode, which can achieve the non-target list qualitative and quantitative detection in a single run, was used for screening all samples. All the MS parameters were optimized to provide the best responses of the analytes. The optimized parameters values are summarized in Section 3.2. 

### 2.3. The Proposed Fragmentation Pathways for 27 β-Blockers

**Figure 3:**
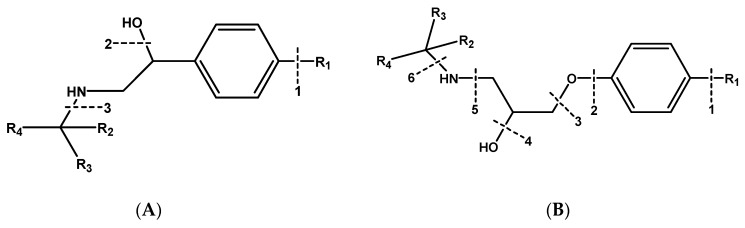
Principal structure of (**A**) phenylethanolamine compounds; (**B**) aryloxypropanolamine compounds.

In this study, secondary mass spectrometry data of all the substances were extracted, and the fragment ions occurring many times were selected to analyze the fragmentation pathway. β-blockers are mainly classified into three kinds of structures. Labetalol and sotalol have the structure of phenylethanolamine, and others have the structure of aryloxypropanolamine except for timolol and hydroxytimolol, which have special chemical structures. The principal structures of phenylethanolamine and aryloxypropanolamine compounds are shown in Figure 3.

#### 2.3.1. Phenylethanolamines Structure

For phenylethanolamine structure, there is a characteristic loss of one molecule of water at position 2 at first, and then a characteristic cleavage at positions 1 and 3, as shown in Figure 3A. This phenomenon was consistent with the fragmentation of β-agonists compounds with the phenylethanolamine structure that were studied in our laboratory [23]. 

#### 2.3.2. Aryloxypropanolamines Structure

For the aryloxypropanolamine structure (Figure 3B), the proposed fragmentation pathways can be divided into three types, depending on the different substitution groups. 

Type I (R_6_ was H, R_7_ and R_8_ were methyl)

**Figure 4:**
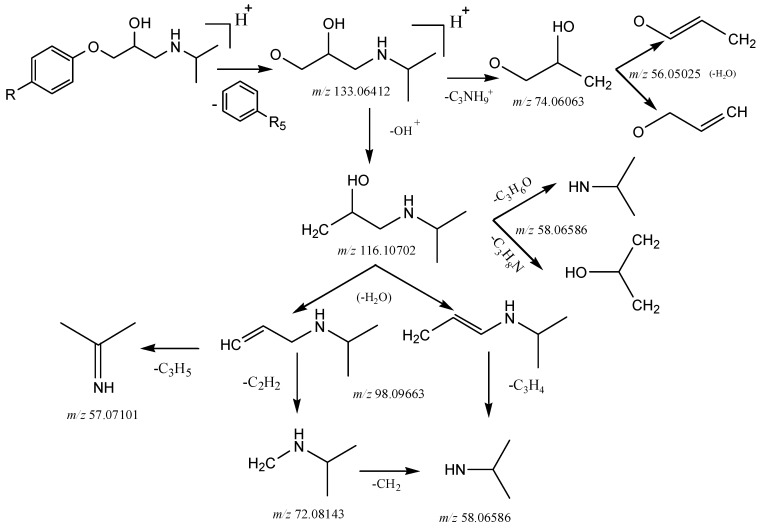
The suggested fragmentation pathways of aryloxypropanolamine compounds.

For type I, the bonds between carbon and oxygen were preferentially broken at position 2. At first, the phenyl structure (C_6_H_4_-R_5_) was lost to form the fragment ion [C_6_H_14_NO_2_ + H]^+^ at *m*/*z* 133.06412. Then, the potential loss of [C_3_NH_9_]^+^ or [OH]^+^ gave *m*/*z* 74.06063 (formula C_3_H_6_O_2_) or 116.10702 (formula C_6_H_14_NO) moieties. And the loss of one molecule of water and a series of fragmentations occurred. The suggested fragmentation pathway of type I is shown in Figure 4.

**Figure 5:**
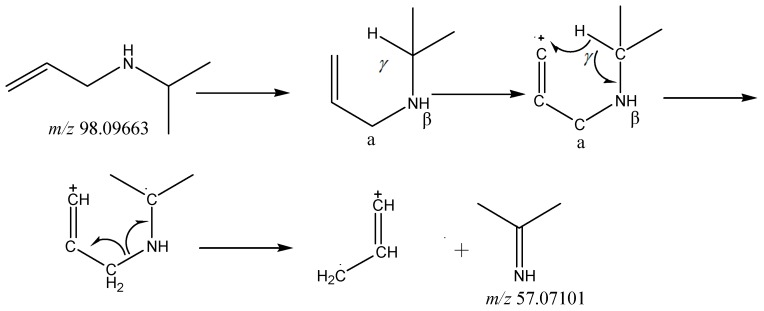
The McLafferty Rearrangement of *m*/z 98.09663.

It is worth mentioning that the Υ-H of the amine structure with 1-propene (C_6_H_12_N at *m*/*z* 98.09663) rearranged to an unsaturated group, accompanied with the cleavage of the β-bond in the amine structure to produce the McLafferty Rearrangement. Imine structure of the fragment C_3_H_7_N (at *m*/*z* 57.07101) was produced by this reaction. The mechanism of the McLafferty Rearrangement reaction is shown in Figure 5.

Type II (R_6_, R_7_ and R_8_ were methyl group)

For type II, the R_6_ position of the aryloxypropanolamine structure was substituted with a methyl group. The methyl group can easily get lost to produce a type I structure, and then have a similar fragmentation pathway to the type I mentioned above. 

Type III (R_6_ or R_7_ was an isophthalic ether structure, others were H)

For type III, the R_6_ or R_7_ position of the aryloxypropanolamine structure was substituted with an isophthalic ether structure, which has strong electronegativity. The bond between carbon and nitrogen at position 8 was easily broken, and it was difficult to form the fragment at *m*/*z* 116.10702 (formula C_6_H_14_NO).

#### 2.3.3. Special Structures

**Figure 6:**
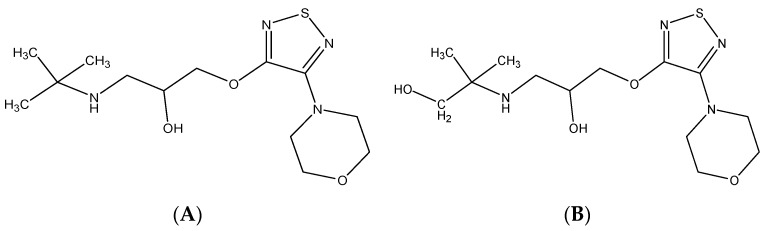
Chemical structures of timolol and hydroxytimolol, (**A**) Timolol, Theoretical *m*/*z* 317.16419 (**B**) Hydroxytimolol, Theoretical *m*/*z* 333.15910.

For timolol and hydroxytimolol (structures see Figure 6.), they can be considered as β-blockers for newly-emerging aryloxypropanolamine compounds, which differ in structure from the others. These two compounds have characteristic fragments at *m*/*z* 74.06063, *m*/*z* 57.07101 and *m*/*z* 56.05025. 

The structure types of the 21 β-blockers and 6 metabolites are listed in Table 1. The possible structures of the corresponding characteristic fragments are described and summarized in Table 2. By exploring the exact mass of these identification markers, it is possible to find newly-emerging β-blockers residues in a complex food matrix.

pαpm=|mmeasured−mtheoretical|mtheoretical×106

### 2.4. The Optimization of the Sample Preparation Procedure

A rapid enzymolysis method was chosen to ensure the processing flux and dissociate the possible bound residual drug. Neutral enzymatic environment (closing to pH 5.2) can significantly reduce matrix co-extraction. Na_2_EDTA was added to the buffer to reduce the chelation between metal ions and strongly polar targets. The alkalized aqueous phase and salting out after the enzymolysis facilitate the target extraction into the organic solvent. pH at 9.0, 9.5, 10.0, 10.5, 11.0 and 12.0 were evaluated, and the best extraction efficiency was obtained at pH 10.0, which was consistent with the literature [1]. 

**Figure 7:**
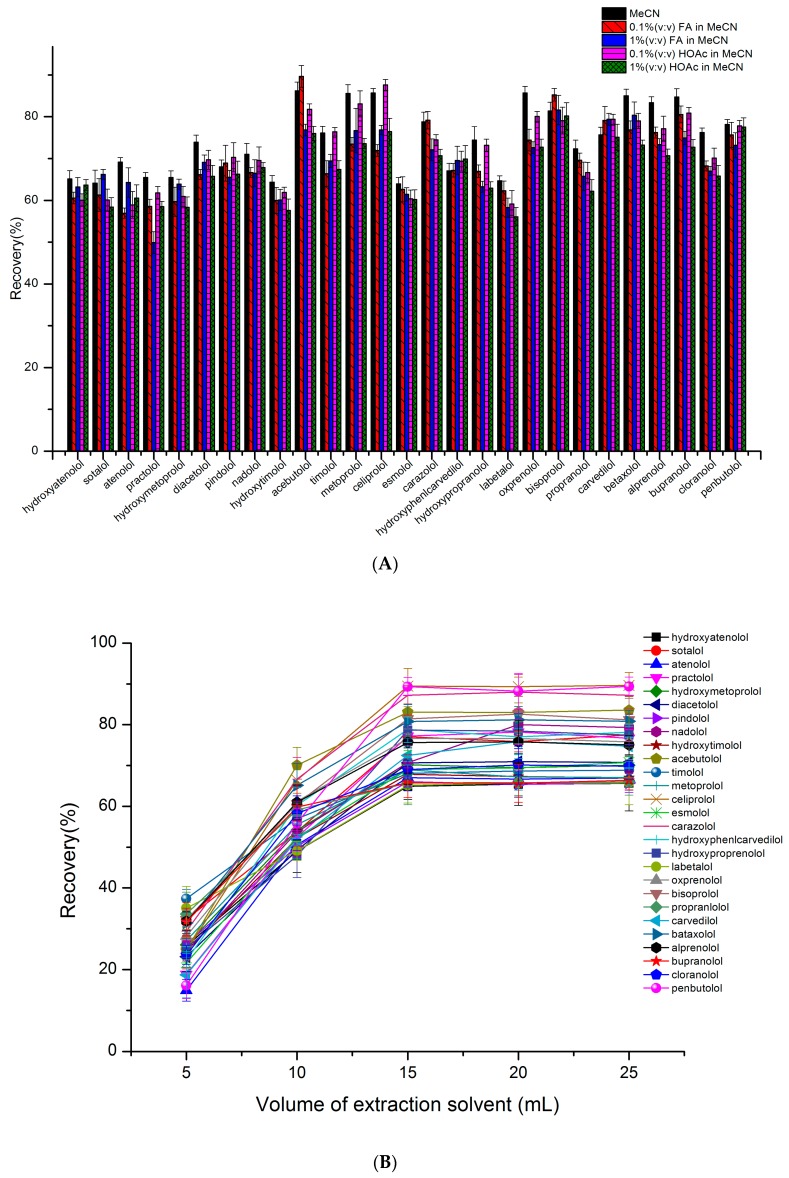
The effect of extracting solvents on the recovery of 27 analytes in milk powder. (**A**) The type of extract solvent, (**B**) the volume of extraction solvent (n = 3).

For food samples, MeCN was commonly used as the extraction solvent due to its protein precipitation ability. Since the acetic-buffer could be used to increase the recoveries of pH-dependent compounds, pure MeCN and different contents of acids (0.1% HOAc, 1% HOAc, 0.1% FA, 1% FA, *v*/*v*) in MeCN were compared for extraction efficiency in this study. For extraction solvents containing HOAc or FA, the recoveries of some analytes (such as sotalol, hydroxymetoprolol, labetalol, epractolol and hydroxytimoloven) were lower than 60%, as can be seen in Figure 7A. Pure MeCN provided better extraction efficiency with all analyte recoveries being higher than 65%; therefore, pure MeCN was found to be the most suitable extract solvent. Then, the solvent volume was investigated for optimization of the recoveries of the targets. It can be observed in Figure 7B that the recoveries of analytes increased with the solvent volume. When the solvent volume reached 15 mL, recoveries began to be stable. In order to ensure the stability of the recoveries, 20 mL extract solvent was chosen to extract all analytes. 

Taking into account the characteristics of the β-blockers and matrix interferences in milk powder samples, low temperature high-speed centrifugation, low temperature high-speed centrifugation + SPE (PRiME HLB column), and low temperature high-speed centrifugation + QuEChERS (quick, easy, cheap, effective, rugged and safe) methods were tested as purification steps. For the low temperature, high-speed centrifugation + QuEChERS method, the water removal step (using anhydrous magnesium sulfate) might take some water-soluble targets (such as sotalol) away, resulting in a low recovery rate. Compared with low temperature, high-speed centrifugation, PRiME HLB column used in SPE step could adsorb nonpolar interferences (some fats and phospholipids) in milk powder samples, which results in a smaller matrix effect and better target recoveries, as shown in Table 3. Therefore, the solid phase extraction PRiME HLB cartridge was selected for purification step.

### 2.5. Validation of the Proposed Method 

#### 2.5.1. Linearity and Sensitivity

The linearity of the proposed method was evaluated using matrix-matched spiked samples over the range of 0.5–500 μg kg^−1^. Calibration curves resulted from the ratios of the peak area of the target compounds to the peak area of the isotope-labeled internal standards. The results showed a good linearity relationship with correlation coefficients (r^2^) higher than 0.995 (Table 4). Limits of detection (LODs) and quantification (LOQs) are fundamental parameters used to evaluate the sensitivity of instructions and methods. The LODs were determined by the injection of a series of diluted standard solutions corresponding to a signal-to-noise (S/N) ratio of 3. The LOQs were determined by the injection of a series of spiked samples corresponding to a signal-to-noise (S/N) ratio of 10. Under the optimum condition, the LODs and LOQs were in the range of 0.2–1.5 μg kg^−1^ and 0.5–5.0 μg kg^−1^, respectively, which allows the quantification of analytes presented at low content, indicating that good sensitivity was obtained. 

#### 2.5.2. Matrix effect

Suppression or enhancement of the target signal usually occurs in the HESI source, especially for complicated food matrices. With a matrix effect (ME) value was between 80% and 120%, signal suppression or enhancement effect can be considered tolerable. As shown in Table 3, many of the analytes did not significantly express the matrix effect, except metoprolol, acebutolol, celiprolol, bisoprolol, diacetolol and α-hydroxyatenolol, which showed a significant matrix effect (ME < 80%, or ME > 120%). In order to accurately quantify the compounds, the assay was quantified with matrix-matched internal standard calibration.

#### 2.5.3. Trueness and Precision

Recovery experiments were performed to evaluate the trueness of the method due to the lack of certified reference materials (CRM). As shown in Table 3, recoveries at three spiking levels (LOQ, 2 × LOQ, 4 × LOQ) ranged from 66.1% to 100.4%. The precision was calculated in terms of intra-day repeatability and inter-day reproducibility, which were expressed as relative standard deviations (RSDs). The results of intra-day and inter-day analyses performed at three spiking levels are presented in Table 3. Repeatability and reproducibility were in the range of 1.6–8.7% and 1.8–8.9%, respectively. Consequently, these results indicated that the developed method in this study is quite reliable, accurate and reproducible for determining β-blockers and their metabolites in milk powder samples.

### 2.6. Real Samples Analysis 

**Figure 8:**
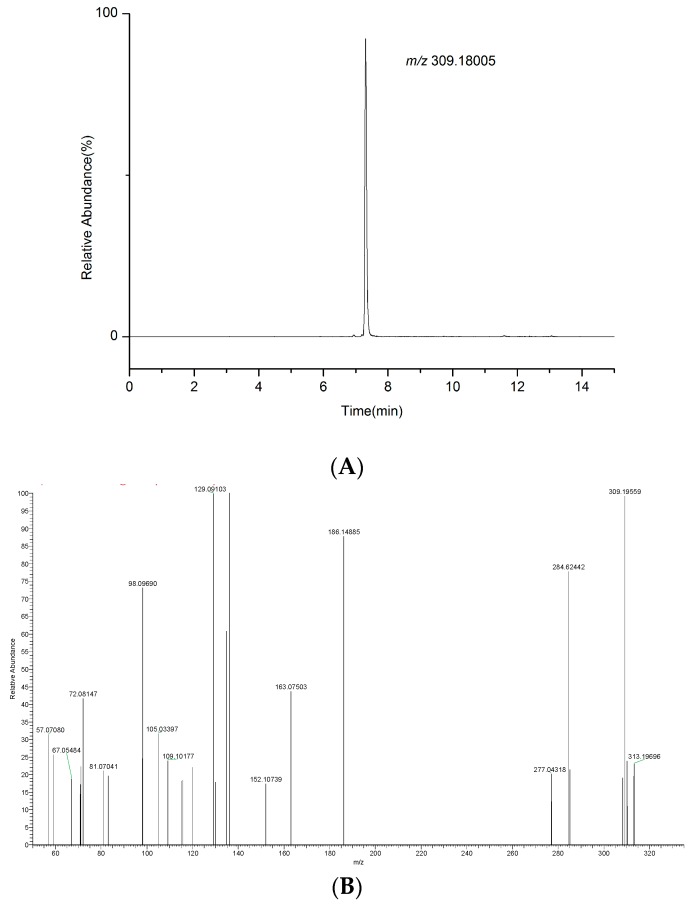
Extracted ion chromatogram and product ion spectrum of the suspected sample. (**A**) The extracted ion chromatogram of the suspected sample. (**B**) The product ion spectrum of the suspected sample.

**Figure 9:**
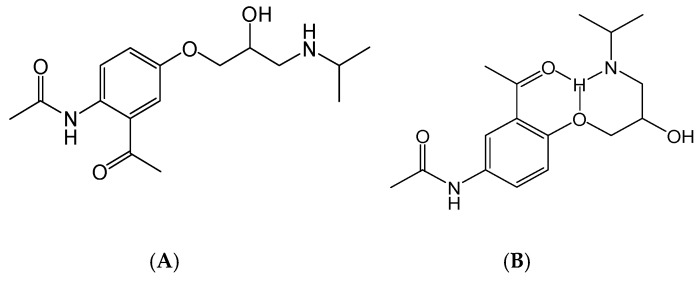
The possible chemical structure of diacetolol isomer detected in sample. (**A**) The first possible chemical structure. (**B**) The second possible chemical structure.

In order to estimate the reliability and practicability of the developed method, 30 samples of infant formula milk powder purchased at local markets were analyzed in this study. The samples were regarded as representative, since they were ranged from phase 1 to stage 4 produced by reputable manufactures. High accuracy parent ions and product ions were used for qualitative analysis simultaneously. Full MS data of this mode were used for quantitative analysis. None of the 27 targeted analytes were detected by the developed Q-Orbitrap high resolution mass spectrometry method. However, an unknown compound which has the same molecule mass (*m*/*z* 309.18005) but different retention time (7.15 min vs. 5.75 min) with diacetolol has been screened in one sample. As shown in Figure 8, under the MS^2^ conditions, the detected unknown compound had high-accuracy product ions 98.09692 (*m*/*z*) and 72.08157 (*m*/*z*), which was similar to diacetolol, and also the characteristic fragment ion *m*/*z* 57.07080. So, it is reasonable to speculate that this unknown substance may be an isomer of diacetolol. Considering the possible structure of product ions, the detected diacetolol’s isomer could have two possible chemical structures (C_16_H_25_O_4_N_2_), as shown in Figure 9. For the first one, the acetyl group on the phenyl ring is in the presence of an acetyl amino group, which is more likely. The reason for this is that intermediate isomers are produced during the synthesis process, resulting in the isomerism of the target compounds. The second possibility may be diastereoisomers. Imino NH, phenolic hydroxyl oxygen and carbonyl oxygen through hydrogen bonding make the nitrogen atom form a relatively stable chiral center, as shown in Figure 9B. The specific structure needs to be further confirmed by NMR or other techniques.

## 3. Materials and Methods

### 3.1. Chemicals and Reagents

Formic acid (FA), acetic acid (HOAc) were purchased from Sigma-Aldrich (Steinheim, Germany). Sodium chloride, ammonium acetate, ethylenediaminetetraacetic acid disodium salt (Na_2_EDTA) were obtained from Beijing Chemical Company (Beijing, China). HPLC-grade methanol (MeOH) and acetonitrile (MeCN) were supplied by Fisher Scientific (Lough borough, UK). Ultra-pure water (H_2_O) was obtained using a Milli-Q Ultrapure system (Millipore, Brussels, Belgium). β-glucuronidase/arylsulfatase was supplied by Merck (Darmstadt, Germany). Solid phase extraction cartridges Oasis PRiME HLB (500 mg, 6 cm^3^) were obtained from Waters (Milford, MA, USA).

Standards of carazolol, timolol maleate, nadolol, sotalol hydrochloride, pindolol, atenolol, metoprolol, carazolol-d_7_ (internal standard, ISTD), propranolol-d_7_ (ISTD) (purity > 96%) were purchased from Dr. Ehrenstorfer (Augsburg, Germany). Acebutolol, carvedilol, penbutolol sulfate, propranolol, betaxolol hydrochloride, alprenolol, oxprenolol, celiprolol, bisoprolol fumarate, labetalol hydrochloride (purity > 96%) were purchased from U.S. Pharma-copoeia (Rockville, MD, USA). Diacetolol, cloranolol, esmolol, bupranolol, practolol, 7-hydroxypropranolol, hydroxytimolol, 4-hydroxyphenylcarvedilol, α-hydroxyatenolol (purity > 96%) were obtained from Toronto Research Chemicals (North York, Canada). α-hydroxymetoprolol (100 μg mL^−1^, methanol) were obtained from AccuStandard (Chiron, Norway). 

All standard stock solutions were prepared in MeOH at 100 μg mL^−1^. The mixed working standard solutions were prepared daily via proportional dilution of the stock solutions. All of the standard solutions were stored at −20 °C in a dark amber bottle.

Matrix-matched standard working solutions were prepared in blank sample extracts, which were obtained from a commercial product purchased from a local market and affirmed in advance not to contain any of the tested analytes. All of the standard solutions were stored at −20 °C in a dark amber bottle.

Extraction solution: 37.5 g Na_2_EDTA was dissolved in an ammonium acetate buffer produced by dissolving 15.4 g ammonium acetate in 1 L deionized water and then using acetic acid to adjust the pH to 5.2.

### 3.2. Instrument and Analytical Conditions

The UHPLC/HESI Q-Orbitrap system consisted of a Thermo UltiMate 3000 UHPLC^+^ system coupled with a Q Exactive mass spectrometer (Thermo Fisher Scientific, Bremen, Germany). The system was controlled by Exactive Tune 1.1 and Xcalibur 2.2 software (Thermo Fisher Scientific, San Jose, CA, USA). 

Chromatographic separation was achieved on an Ascentis^®^ C8 chromatographic column (100 × 4.6 mm, 3 μm) (SUPELCO^®^ Analytical, Bellefonte, PA, USA). The autosampler tray temperature, column oven temperature, flow rate and injection volume were set at 10 °C, 30 °C, 0.5 mL min^−1^ and 2 μL, respectively. The mobile phase consisted of water containing 0.1% FA (A) and MeOH (B). The gradient used for eluting analytes with mobile phase is as follows: 0–0.5 min, 5% B; 0.5–9 min, 5–95% B; 9–12.5 min, 95% B; 12.5–14 min, 95–5% B; 14–15 min, 5% B.

The Q-Orbitrap HRMS was equipped with a heated electrospray ionization (HESI, Waltham, MA, USA) source and the analysis was operated in the Full MS/dd-MS^2^ (data-dependent MS^2^) scanning mode, which includes a Full scan followed by MS/MS scan of precursors in the inclusion list. All analytes were measured in positive mode and precursor ion selected was [M + H]^+^ in all cases. To keep a balance between the selectivity and the sensitivity with Full MS, a mass resolution of 70,000 FWHM was selected, and this turned out to be optimal for the majority of the analytes. For the dd-MS^2^ scan, 35,000 FWHM was used for time-saving and to ensure sufficient scan points of the Full MS. The stepped normalized collision energy (NCE) was set to 15%, 25% and 35%. The spray voltage, capillary temperature, aux gas heater temperature were set to 3.0 kV, 350 °C and 350 °C, respectively. The sheath gas, auxiliary gas, sweep gas and S-lens RF level were set to 40, 10, 0 (arbitrary units) and 50 V, respectively. Main MS acquisitions parameters are listed in Table 1. All the extracted mass traces were based on a 2 ppm mass window (accuracy). 

### 3.3. Sample Preparation

Two grams of each sample were precisely weighed in polypropylene centrifuge tube (50 mL). Then, 100 μL mixed ISTD solution (1μg mL^−1^), 40 μL β-glucuronidase/arylsulfatase and 5 mL EDTA extract solution were added to the sample after being fully dissolved in 5 mL H_2_O. The mixture was placed in a water bath shaker at 50 °C for 60 min after vortex-mixing for 1 min. After cooling to room temperature, the pH of the extract was adjusted to 10.0 with 3 mol L^−1^ NaOH solution. MeCN (20 mL) and NaCl (2.5 g) were added to the mixture and then shaken for 30 min. After that, the extract was centrifuged at 10,000 rpm at 4 °C for 10 min. The supernatant was decanted to another polypropylene centrifuge tube. The above procedure was repeated and combined with the supernatant. Next, 2 mL of the supernatant directly passed through the Oasis PRiME HLB (500 mg, 6 cm^3^) cartridge. After sample loading, the cartridge was washed with 2 mL H_2_O/MeOH (95:5, *v*/*v*) and 2 mL MeOH/MeCN (1:9, *v*/*v*), and all of the effluent was collected. The mixture was evaporated with a gentle N_2_ stream at 40 °C, and redissolved in 1 mL of H_2_O/MeOH (1:1). The sample extract was vortexed for 0.5 min and filtered through a 0.22 μm nylon membrane, and was ready for Q-Orbitrap HRMS analysis.

### 3.4. Method Validation

Linearity, precision and recovery were carried out to validate the method. An internal standard method which using carazolol-d_7_ and propranolol-d_7_ as ISTD was utilized for quantification. A matrix-matched calibration cure was constructed by linear regression of the ratios of chromatographic peak areas of the standards and the ISTD. The linearity was discussed by the coefficient of determination (r^2^). 

Blank milk sample powders spiked at three concentration levels (LOQ, 2 × LOQ and 4 × LOQ) which were tested for the recovery experiments. Each level was analyzed in five replicates. Intra-day precision was performed by spiking blank milk at three concentration levels (LOQ, 2 × LOQ and 4 × LOQ) with five replicates in one day. To evaluate inter-day precision, the same concentration levels were performed during over consecutive days.

The matrix effect (ME) was calculated by comparing the response of analytes prepared in solvent and in extracted blank matrix at the same concentration, respectively. The value of matrix effect can be calculated as (Equation (1)):

(1) ME(%)=B/A×100

A refers to the peak areas obtained from neat solution standards, while B refers to the corresponding peak areas of standards spiked after extraction from matrix [24,25].

## 4. Conclusions

In this study, a rapid HPLC-Q-Orbitrap HRMS method for simultaneous analyses of 27 compounds (21 β-blockers and 6 metabolites) in milk powder has been developed. Simultaneous qualitative and quantitative analysis of analytes were achieved using Full MS/dd-MS^2^ acquisition mode of the Q-Orbitrap mass analyzer and the preparation procedure comprised a simple acetonitrile step, followed by a cleanup using cartridges. The method has been well validated, and is particularly effective and valuable for the routine screening of β-blockers and metabolites in infant formula milk powder. At the same time, the corresponding characteristic fragmentation behavior of the 27 compounds were explored, the characteristic product ions were determined and applied to the actual sample screening.

## Figures and Tables

**Table 1:** Formula, ionization mode, theoretical mass, measured mass, mass accuracy and MS^2^ data for 27 β-blockers.

Analytes	Formula	Theoretical Mass [*m*/*z*]	Measured Mass [*m*/*z*]	Accuracy ^α^ [ppm]	MS^2^	Structure Type
Carazolol	C_18_H_22_N_2_O_2_	299.17540	299.17484	1.87	222.09090 116.10712 98.09675 72.08148 56.05032	Type I
Oxprenolol	C_15_H_23_NO_3_	266.17507	266.17496	0.41	133.06451 116.10696 98.09662 72.08138 56.05027	Type I
Propranolol	C_16_H_21_NO_2_	260.16451	260.16373	3.00	183.07979 116.10689 98.09659 72.08135 58.06586	Type I
Alprenolol	C_15_H_23_NO_2_	250.18016	250.17953	2.52	173.09550 116.10687 98.09660 72.08134 56.05024	Type I
Bisoprolol	C_18_H_31_NO_4_	326.23258	326.23169	3.00	133.06441 116.10689 98.09659 74.06059 56.05026	Type I
Betaxolol	C_18_H_29_NO_3_	308.22202	308.22174	0.91	133.06445 116.10691 98.09661 72.08135 56.05004	Type I
Sotalol	C_12_H_20_N_2_O_3_S	273.12674	273.12680	0.46	255.11484 213.06822 176.12991 133.07552 198.05713	phenylethanolamine
Pindolol	C_14_H_20_N_2_O_2_	249.15975	249.15961	0.56	172.07532 116.10711 98.09679 72.08147 58.06597	Type I
Nadolol	C_17_H_27_NO_4_	310.20128	310.20084	1.42	354.13795 236.12750 201.09059 74.06068 56.05030	Type II
Timolol	C_13_H_24_N_4_O_3_S	317.16419	317.16367	1.64	261.10089 244.07440 188.04840 74.06068 57.07074	Special structure
Acebutolol	C_18_H_28_N_2_O_4_	337.21218	337.21310	2.73	218.11726 116.10712 98.09680 72.08150 56.05036	Type I
Celiprolol	C_20_H_33_N_3_O_4_	380.25438	380.25299	3.67	324.19070 307.16397 251.10155 74.06061 56.05026	Type II
Labetalol	C_19_H_24_N_2_O_3_	329.18597	329.18613	0.49	311.17395 294.14755 207.11201 179.08063 162.05423	phenylethanolamine
Cloranolol	C_13_H_19_Cl_2_NO_2_	292.08656	292.08658	0.07	236.02318 218.01273 174.97054 74.06063 56.05020	Type II
Penbutolol	C_18_H_29_NO_2_	292.22711	292.22672	1.33	236.16374 201.12683 133.06451 74.06063 57.07070	Type II
Practolol	C_14_H_22_N_2_O_3_	267.17032	267.16965	2.51	190.08589 116.10711 98.09682 72.08146 56.05036	Type I
Carvedilol	C_24_H_26_N_2_O_4_	407.19653	407.19565	2.16	283.14340 224.12755 100.07599 74.06063 56.05036	Type III
Bupranolol	C_14_H_22_ClNO_2_	272.14118	272.14020	3.60	216.07790 198.06741 181.04089 74.06061 56.05027	Type II
Atenolol	C_14_H_22_N_2_O_3_	267.17032	267.16983	1.83	133.06412 116.10690 98.09663 74.06060 56.05026	Type I
Esmolol	C_16_H_25_NO_4_	296.18563	296.18558	0.17	133.06467 116.10737 98.09705 72.08168 56.05050	Type I
Metoprolol	C_15_H_25_NO_3_	268.19072	268.19028	1.64	133.06435 116.10693 98.09660 74.06057 56.05026	Type I
Diacetolol	C_16_H_24_N_2_O_4_	308.18088	308.18088	0.00	291.16943 116.10702 98.09670 72.08143 56.05031	Type I
α-hydroxymetoprolol	C_15_H_25_NO_4_	284.18563	284.18472	3.20	133.06435 116.10691 98.09663 74.06059 56.05026	Type I
α-hydroxyatenolol	C_14_H_22_N2O_4_	283.16523	283.16507	0.57	133.08632 116.10760 89.06059 74.06103 57.07010	Type I
(*S*)-Hydroxytimolol	C_13_H_24_N_4_O_4_S	333.15910	333.15823	2.61	261.10059 188.04814 146.11705 74.06059 56.05025	Special structure
7-Hydroxyproprenolol	C_16_H_21_NO_3_	276.15942	276.15930	0.43	199.07463 116.1067 98.09663 74.06057 58.06586	Type I
4-Hydroxyphenylcarvedilol	C_24_H_26_N_2_O_5_	423.19145	423.19141	0.09	283.14267 240.12180 100.07578 74.06049 56.05022	Type III

**Table 2:** The possible structure of corresponding characteristic fragments.

No.	*m*/*z*	The Molecular Formula	The Possible Structure
**1**	56.05025	C_3_H_4_O	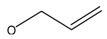 or 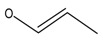
**2**	57.07101	C_3_H_7_N	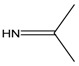
**3**	58.06586	C_3_H_6_O or C_3_H8N	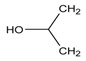 or 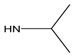
**4**	72.08143	C_4_H_10_N	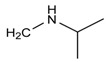
**5**	74.06063	C_3_H_6_O_2_	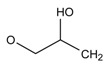
**6**	98.09663	C_6_H_12_N	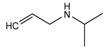 or 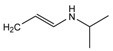
**7**	116.10702	C_6_H_14_NO	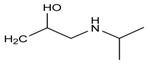
**8**	133.06412	C_6_H_15_NO_2_^+^	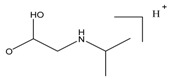

**Table 3:** Validation parameters of the developed method.

NO.	Analyte	Matrix Effect C (%)		QC Concentration (μg kg^−1^)	Average Recovery (%)	Intra-Day Precision (%) (n = 5)	Inter-Day Precision (%) (n = 5)
		PRiME HLB	Centrifugation				
**1**	Atenolol	115.7	118.5	2	72.5	2	3.1
				4	76.3	3.1	2.2
				8	74.8	4.1	5
**2**	Sotalol	85.6	86.8	2	83.6	3.2	3.2
				4	87	5.6	1.9
				8	81.1	2.7	7.9
**3**	Pindolol	101.7	108.2	1	89.2	1.6	5.2
				2	100.4	4.8	2.7
				4	83.7	3.6	6.4
**4**	Nadolol	102.2	112.8	0.5	83.6	7.9	5.5
				1	93.2	2.1	3.1
				2	78.5	4.4	5.3
**5**	Metoprolol	120.9	140	1	80.4	3.9	5
				2	90	6.2	3.5
				4	84.2	2.1	7.7
**6**	Timolol	116.9	133.7	1	76.5	2.1	7.4
				2	83.8	5.2	3.9
				4	78.8	3.8	3.2
**7**	Acebutolol	129.2	155.3	0.5	95.6	2.2	3.2
				1	89.3	5.7	5
				2	92.1	2.4	7.5
**8**	Oxprenolol	109.9	123.3	1	69.6	1.9	4.4
				2	89.7	6.7	7.5
				4	84.8	3.3	3.9
**9**	Celiprolol	165.1	181.5	1.5	98.5	7.3	5.6
				3	87.4	3.5	5.4
				6	93.3	2.1	7.7
**10**	Bisoprolol	134.6	156.3	0.5	93.8	7.1	7.2
				1	90.4	4.4	3.6
				2	84.6	2.5	5.6
**11**	Labetalol	91.2	102.1	0.5	91.7	2.1	2.8
				1	86.6	7.5	6.4
				2	83.4	3.3	6.5
**12**	Alprenolol	102.1	117	0.5	74.8	5.5	4.2
				1	81.3	7.2	8.9
				2	81.1	4.1	5.4
**13**	Propranolol	98.5	120.7	0.5	80.2	8.7	2.3
				1	83.5	3.5	1.8
				2	80.4	6.4	5.6
**14**	Betaxolol	117.6	146.5	2	79.8	7.1	4.5
				4	91.5	2.7	3.7
				8	85.2	5.6	2.5
**15**	Cloranolol	109.2	126.1	2	72.1	8.1	3.5
				4	75.5	3.5	5.4
				8	76.6	3.1	7.2
**16**	Penbutolol	109.4	134.3	1	85.5	3.4	6.5
				2	97.6	2.6	4.4
				4	76.4	1.7	2.5
**17**	Practolol	115.7	120.6	0.5	71.9	4.4	2.6
				1	73.6	3.4	7.5
				2	75.2	5	5.8
**18**	Carazolol	80.1	96.1	0.5	99.3	3.1	2.8
				1	85.3	4	5.7
				2	85.9	7.9	4.9
**19**	Carvedilol	82.7	105.6	2	79.5	5.7	2.5
				4	78	2.5	7.5
				8	84.6	3.4	5.6
**20**	Esmolol	101.8	106.9	3	72.5	5.1	3.6
				6	83.2	8.1	2.3
				12	73.4	4.3	7.5
**21**	Bupranolol	112.6	122.9	0.5	73.4	3.2	5.9
				1	79.4	5.2	3.5
				2	80	1.7	5.4
**22**	Diacetolol	134	152.2	1	81.7	3.8	3.5
				2	87.7	2.3	5.9
				4	82.9	6.9	2.5
**23**	α-Hydroxymetoprolol	107.5	113.8	1	85.8	2.4	7.1
				2	89.8	4.7	4.6
				4	84.5	5.6	5.6
**24**	α-Hydroxyatenolol	80.1	72.8	5	67.7	4.3	3.2
				10	66.1	3.4	5.4
				20	68.6	7.7	6.9
**25**	(s)-Hydroxytimolol	93.4	103.6	1	78.5	2.2	5.8
				2	91.3	5.5	5.7
				4	85.7	6.3	4.6
**26**	7-Hydroxypropranolol	84.7	99.9	1	69.8	5.6	3.5
				2	73.6	2.2	5.3
				4	78.8	3.2	7.2
**27**	4-Hydroxyphenlcarvedilol	85.6	103.1	2	73.5	3.7	1.8
				4	66	7	4.5
				8	67.4	6.3	6.9

**Table 4:** Regression data, Precision, LODs, and LOQs for the investigated compounds.

Analytes	Linear Equation	Linear Range (μg kg^−1^)	Correlation Coefficient (r^2^)	LOD (μg kg^−1^)	LOQ (μg kg^−1^)
Atenolol	Y = −0.0196094 + 0.0399798X	2–200	0.9994	0.6	2
Sotalol	Y = −0.0302291 + 0.0364729X	2–200	0.9995	0.6	2
Pindolol	Y = 0.374297 + 0.109209X	1–200	0.9967	0.3	1
Nadolol	Y = 0.00696148 + 0.0330124X	0.5–50	0.9987	0.2	0.5
Metoprolol	Y = 0.250919 + 0.0925317X	0.5–50	0.9975	0.3	1
Timolol	Y = −0.0667935 + 0.0862132X	1–100	0.9997	0.3	1
Acebutolol	Y = 0.0484461 + 0.0478361X	0.5–50	0.9977	0.2	0.5
Oxprenolol	Y = −0.157711 + 0.0272438X	1–100	0.9990	0.3	1
Celiprolol	Y = −0.00691133 + 0.068192X	2–200	0.9966	0.5	1.5
Bisoprolol	Y = 0.130461 + 0.111734X	0.5–50	0.9969	0.2	0.5
Labetalol	Y = −0.0396352 + 0.0389041X	0.5–50	0.9997	0.2	0.5
Alprenolol	Y = −0.164222 + 0.588251X	0.5–50	0.9997	0.2	0.5
Propranolol	Y = 0.0870785 + 0.148381X	0.5–50	0.9996	0.2	0.5
Betaxolol	Y = 0.132601 + 0.130952X	2–200	0.9987	0.6	2
Cloranolol	Y = 0.214191 + 0.103506X	2–200	0.9991	0.6	2
Penbutolol	Y = −0.0422939 + 0.063028X	1–100	0.9989	0.3	1
Practolol	Y = −0.0153134 + 0.114317X	0.5–50	0.9990	0.2	0.5
Carazolol	Y = −0.015998 + 0.0807354X	0.5–50	0.9998	0.2	0.5
Carvedilol	Y = −0.00662109 + 0.0671112X	2–200	0.9998	0.6	2
Esmolol	Y = −0.142646 + 0.159519X	5–500	0.9994	1	3
Bupranolol	Y = −0.126666 + 0.325598X	0.5–50	0.9995	0.3	0.5
Diacetolol	Y = 0.204209 + 0.0797893X	1–100	0.9973	0.3	1
α-Hydroxymetoprolol	Y = 0.0134667 + 0.105121X	1–100	0.9992	0.3	1
α-Hydroxyatenolol	Y = −0.0738747 + 0.0396782X	5–500	0.9993	1.5	5
(*S*)-Hydroxytimolol	Y = 0.168441 + 0.119248X	1–100	0.9989	0.3	1
7-Hydroxypropranolol	Y = 0.0104 + 0.167864X	1–100	0.9999	0.3	1
4-Hydroxyphenlcarvedilol	Y = 0.0993695 + 0.159881X	2–200	0.9991	0.6	2

Y: The ratio of the peak area of the target to the area of the isotope peak, X: Corresponding concentration (μg kg^−1^).
